# Effectiveness of ChatGPT 4.0 in Telemedicine-Based Management of Metastatic Prostate Carcinoma

**DOI:** 10.3390/diagnostics14171899

**Published:** 2024-08-29

**Authors:** Emre Dal, Ayana Srivastava, Beverly Chigarira, Chadi Hage Chehade, Vinay Matthew Thomas, Gliceida M. Galarza Fortuna, Diya Garg, Richard Ji, Georges Gebrael, Neeraj Agarwal, Umang Swami, Haoran Li

**Affiliations:** 1 Huntsman Cancer Institute, University of Utah, Salt Lake City, UT 84112, USA; u1413765@utah.edu (E.D.); ayana.srivastava@hci.utah.edu (A.S.); beverly.chigarira@hci.utah.edu (B.C.); chadi.chehade@hci.utah.edu (C.H.C.); vinay.mathewthomas@hci.utah.edu (V.M.T.); gliceida.galarzafortuna@hsc.utah.edu (G.M.G.F.); u6037707@utah.edu (D.G.); richard.ji@hci.utah.edu (R.J.); georges.gebrael@hsc.utah.edu (G.G.); neeraj.agarwal@hci.utah.edu (N.A.); umang.swami@hci.utah.edu (U.S.); 2 Department of Medical Oncology, University of Kansas Cancer Center, Westwood, KS 66205, USA

**Keywords:** artificial intelligence, ChatGPT, metastatic prostate cancer, telemedicine, virtual clinic

## Abstract

The recent rise in telemedicine, notably during the COVID-19 pandemic, highlights the potential of integrating artificial intelligence tools in healthcare. This study assessed the effectiveness of ChatGPT versus medical oncologists in the telemedicine-based management of metastatic prostate cancer. In this retrospective study, 102 patients who met inclusion criteria were analyzed to compare the competencies of ChatGPT and oncologists in telemedicine consultations. ChatGPT’s role in pre-charting and determining the need for in-person consultations was evaluated. The primary outcome was the concordance between ChatGPT and oncologists in treatment decisions. Results showed a moderate concordance (Cohen’s Kappa = 0.43, *p* < 0.001). The number of diagnoses made by both parties was not significantly different (median number of diagnoses: 5 vs. 5, *p* = 0.12). In conclusion, ChatGPT exhibited moderate agreement with oncologists in management via telemedicine, indicating the need for further research to explore its healthcare applications.

## 1. Introduction

Initially conceptualized as a means to bridge the geographical gap between patients and healthcare providers, telemedicine has evolved with technological advancements. Integrating electronic communication means to facilitate medical practice has fundamentally altered the reach of healthcare services [1]. This evolution was notably accelerated by the COVID-19 pandemic, which necessitated remote healthcare practices to maintain patient and healthcare worker safety [2]. Modern telemedicine now includes a variety of services, such as video consultations, electronic health records, remote monitoring of vital signs, and digital diagnostic tools, reflecting its broadened scope beyond mere telephonic or video consultations [3].

Integrating Artificial Intelligence (AI) in telemedicine is a transformative development. AI in healthcare, particularly through machine learning, natural language processing, and deep learning, is enhancing diagnostic precision, personalizing patient care, and optimizing clinical workflows [4]. ChatGPT 4.0, an advanced AI language model developed by OpenAI, exemplifies this integration. With its ability to understand and generate human-like text, ChatGPT 4.0 is poised to be used in various healthcare applications, from triaging patient queries to assisting in generating medical documentation and providing decision support for clinicians [5]. Tools like ChatGPT have the potential to significantly improve the efficiency and quality of telemedicine services, especially in managing chronic conditions or in specialties like oncology, where nuanced and individualized treatment planning is critical [6].

Recent studies highlight the growing applications of AI in various medical fields, demonstrating its potential to revolutionize telemedicine and enhance patient care. Explainable AI has shown promise in improving diagnostic accuracy and aiding surgical procedures, allowing for more transparent and reliable decision-making processes in clinical settings [7]. Advancements in machine learning models have significantly improved the classification of medical images, such as those used in breast cancer diagnostics, leading to earlier and more accurate detection of malignancies [8]. Additionally, the development of quantum iterative reconstruction techniques for low-dose, ultra-high-resolution photon-counting detector CT of the lung enhances the quality of diagnostic imaging while minimizing radiation exposure [9]. These advancements collectively underscore the transformative potential of AI in telemedicine, paving the way for more precise, efficient, and accessible healthcare services.

Complementing these developments, recent advancements in artificial intelligence, particularly in natural language processing, have positioned tools like ChatGPT at the forefront of medical AI applications. Research by Sallam [10] has shown that ChatGPT can greatly enhance the efficiency of clinical documentation, automating routine tasks and aiding in medical decision-making processes. Deng and Lin [11] further highlight the benefits and challenges of ChatGPT, noting its significant potential in improving patient engagement through conversational interfaces that have proven effective in triaging patient queries and providing initial medical advice. Stokel-Walker and Van Noorden [12] explored the broader implications of generative AI in science, emphasizing ChatGPT’s role in telemedicine platforms, revealing how it can streamline virtual consultations and assist clinicians in managing chronic diseases more effectively. Mann’s [13] interview on artificial intelligence in translational medicine underscores ChatGPT’s proficiency in generating coherent and contextually accurate medical records, significantly reducing the administrative burden on healthcare providers. Additionally, Marchandot et al. [14] discuss the role of ChatGPT in academic writing for cardiologists, highlighting both its potential benefits and ethical dilemmas, underscoring the importance of continuous medical education where ChatGPT serves as an interactive tool for training healthcare professionals. These studies collectively illustrate the transformative potential of ChatGPT in enhancing various aspects of telemedicine, from improving patient interactions to optimizing clinical workflows.

There is considerable interest in assessing the performance of ChatGPT in managing complex medical conditions, including metastatic prostate carcinoma (mPC) [15], selected for its chronic nature and slow disease progression, and the need for nuanced, long-term care strategies, making it an ideal case for evaluating the potential of AI in enhancing patient management and treatment outcomes [16].

While AI’s role in diagnostics, patient engagement, and administrative efficiency has been studied [5], its effectiveness in managing complex conditions like mPC through telemedicine remains underexplored. This study aimed to compare the effectiveness of ChatGPT 4.0 and medical oncologists in the telemedicine management of mPC.

The primary objectives of this study are to investigate the ability of ChatGPT 4.0 to determine the continuation or cessation of current treatment in patients with metastatic prostate cancer seen via telemedicine and to evaluate the clinical decision-making complexity of ChatGPT 4.0, using mini-Clinical Evaluation Exercise (miniCEX), in comparison to medical oncologists. This study hypothesizes that ChatGPT 4.0 can demonstrate comparable efficacy to medical oncologists in determining the need to continue or discontinue current treatment for patients with metastatic prostate cancer in a telemedicine context, and that ChatGPT 4.0 can effectively utilize pre-charting information to generate relevant questions for a virtual visit and formulate an appropriate management plan, with decision-making complexity comparable to that of medical oncologists.

## 2. Materials and Methods

### 2.1. Participants

This study examined a cohort of 102 patients with mPC treated at the Huntsman Cancer Institute, University of Utah, from 1 April 2022 to 30 March 2023. These patients exhibited various stages of metastasis and underwent diverse therapeutic regimens, reflecting the real-world complexities of clinical practice. Data for this study were gathered through a comprehensive review of electronic medical records (EMRs) at the Huntsman Cancer Institute. The collection process involved accessing and compiling deidentified patient information, including telemedicine consultation notes, preceding clinical notes, laboratory results, imaging studies, and treatment plans.

### 2.2. Study Design

This investigation is conducted as an Institutional Review Board (IRB)-approved retrospective study, systematically analyzing historical patient records and AI interaction data to evaluate treatment outcomes and technology integration in healthcare. In this proof-of-concept study, we hypothesized that ChatGPT can be utilized to prescreen and analyze the data from patients with mPC at telemedicine clinics and determine who are at an increased risk of complications that need to be managed in person.

### 2.3. Inclusion and Exclusion Criteria

Patients were included in the study if they had a confirmed diagnosis of metastatic prostate cancer. They should have participated in at least one telemedicine consultation. The availability of comprehensive documentation from at least two consecutive telemedicine visits was necessary to analyze treatment decisions and changes over time comprehensively. Conversely, patients were excluded if they had a diagnosis of non-metastatic localized prostate cancer. Patients who had only in-person visits were also excluded.

### 2.4. Data Collection

Data for this study were gathered through a comprehensive review of electronic medical records (EMRs) at the Huntsman Cancer Institute. The collection process involved accessing and compiling detailed patient information, including telemedicine consultation notes, preceding clinical notes, laboratory results, imaging studies, and treatment plans. 

**Figure 1:**
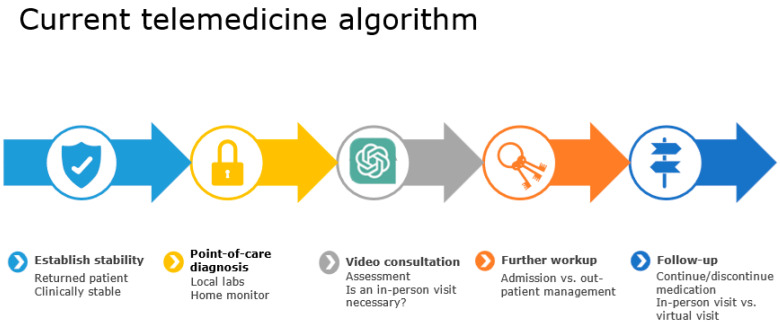
Study algorithm.

The patient’s chart from the previous visit, updated point-of-care lab, and image results were uploaded to ChatGPT blindly in a HIPPA-compliant manner. ChatGPT was tasked with determining whether the patient could be safely managed in a virtual visit or if modifications to the current treatment plan were necessary. The primary outcome is the decision made by ChatGPT if a face-to-face consultation is needed. Then, the note generated by ChatGPT was compared to the oncologist’s note. The mini-Clinical Evaluation Exercise (miniCEX) scores and medical decision-making (MDM) complexity ratings were used to assess and compare the clinical performance and decision-making complexity between ChatGPT and the oncologists (Figure 1). In the interim, the duration spent on charting by the oncologist was recorded and utilized to compare the time spent by ChatGPT, as well as to assess the potential time saved if ChatGPT was employed to undertake the same charting task.

### 2.5. Statistical Analysis

The concordance between ChatGPT and oncologists was assessed using Cohen’s Kappa test. The Mann–Whitney U test was employed to compare the two groups’ miniCEX and MDM complexity scores. The length of MDM in the consultation note provided by ChatGPT and medical oncologists was compared using a paired *t*-test. Statistical analyses were conducted using the R software package 4.3.3. In all analyses, a *p*-value of less than 0.05 was considered statistically significant.

## 3. Results

### 3.1. Demographic and Clinical Baseline Characteristics

The median age was 75 years (range: 53 to 99 years). The racial composition included White (97.06%), Hispanic (1.96%), and Hawaiian or Pacific Islander populations (0.98%). Regarding disease-specific metrics, the median Gleason score was 7, ranging from 6 to 10. Most patients (54.84%) had an ECOG Performance Status score of 1. Bone (47.40%) and lymph nodes (44.16%) were the most common metastatic sites. The most common comorbid conditions included hypertension (28.92%), hyperlipidemia (24.56%), and gastroesophageal reflux disease (18.13%) (Table 1).

### 3.2. Evaluation of AI Performance in Treatment Decision-Making

#### 3.2.1. Assessment of Concordance in Treatment Decisions between ChatGPT 4.0 and Medical Oncologists for Metastatic Pancreatic Cancer

**Figure 2:**
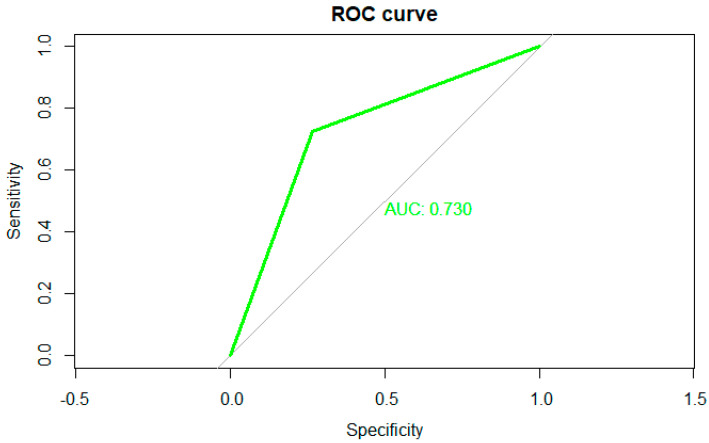
ROC curve of concordance between ChatGPT and medical oncologist. (Green line: calculate ROC curve; Grey line: chance level).

The concordance between ChatGPT 4.0 and medical oncologists in the context of treatment decisions for mPC showed a moderate agreement level (Kappa = 0.43, *p* < 0.001) (Figure 2), which suggests there was still a significant amount of discordance that could impact clinical decision-making.

The figure presents a Receiver Operating Characteristic (ROC) curve, a tool used to assess the performance of a binary classifier system. The curve plots sensitivity (true positive rate) on the *y*-axis against 1—specificity (false positive rate) on the *x*-axis, illustrating the trade-off between these metrics as the classifier’s decision threshold varies. The green line represents the ROC curve, indicating the classifier’s ability to differentiate between positive and negative classes, while the diagonal gray line reflects the performance of a random classifier with an area under the curve (AUC) of 0.5. The AUC for this classifier is 0.730, suggesting a moderate discriminative capacity. Thus, an AUC of 0.730 demonstrates that the classifier can reasonably distinguish between the classes, although there is room for improvement.

#### 3.2.2. Comparative Evaluation of Diagnostic Accuracy, Sensitivity, and Specificity of ChatGPT versus Oncologists in Telemedicine Consultations

Sensitivity measures the AI’s ability to correctly identify cases requiring a change in treatment, while specificity assesses its accuracy in confirming cases that do not necessitate such changes. There was statistical significance between the decision-making of ChatGPT and that of clinicians (Chi-squared = 5.1, *p* = 0.02). 

**Figure 3:**
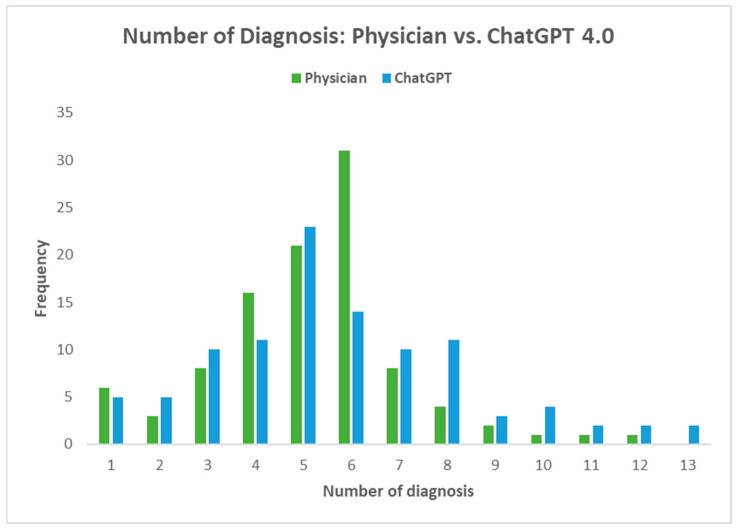
Number of diagnoses made by ChatGPT versus medical oncologist.

The number of diagnoses made by ChatGPT compared to oncologists did not differ significantly (median number of diagnoses: 5 vs. 5, *p* = 0.12) (Figure 3). Additionally, the median miniCEX score for ChatGPT was high (median score = 8, out of 10), indicating proficient clinical performance in simulated educational environments (Table 2).

A notable finding was the average time saved by ChatGPT in pre-charting (charting time: 42 min by oncologist vs. 1 min by ChatGPT). This significant reduction in preparatory work could suggest that AI integration may enhance the efficiency of telemedicine by freeing up clinicians’ time for direct patient interaction or managing a large patient load.

## 4. Discussion

In this study, ChatGPT displayed moderate concordance compared to oncologists in clinical decision-making when managing patients with mPC in telemedicine. Additionally, the study found no significant difference in the number of diagnoses made by ChatGPT compared to oncologists, highlighting ChatGPT’s capability to make accurate clinical diagnoses. Furthermore, ChatGPT demonstrated a high level of efficiency, as evidenced by its median miniCEX score of 8 and a median MDM length of 41 words. The utilization of ChatGPT in clinical settings resulted in an average time saving of 41 min in pre-charting activities, indicating its potential to significantly streamline clinical workflows and improve the efficiency of patient care processes.

### 4.1. Interpretation of Results

The moderate concordance between ChatGPT 4.0’s recommendations and the decisions made by medical oncologists carries substantial implications for the integration of AI into clinical practice. This level of agreement suggests that AI has the potential to replicate some aspects of an oncologist’s clinical reasoning, which is a noteworthy advancement in the field of medical AI [17]. However, the findings also highlight the current limitations of AI in fully understanding the nuances and complexities of medical decision-making that are often second nature to experienced clinicians. Currently, while ChatGPT 4.0 can provide valid input, it is not a substitute for human judgment. Instead, it should be viewed as a decision-support tool that can contribute to a more streamlined healthcare process, especially in a telemedicine setting where rapid assessments are often needed [18]. 

The assessment of ChatGPT 4.0’s clinical competence using the miniCEX and MDM highlights the AI’s proficiency in certain areas of clinical evaluation and decision-making. The miniCEX scores, with a median of 8, suggest that the AI’s clinical reasoning and documentation meet high standards, at least in a structured environment with predefined parameters. Meanwhile, the median MDM length indicates a concise and focused approach in decision-making documentation, which may reflect efficiency in communication, though it lacks the depth a human clinician might provide.

The implications of these performance metrics are twofold. Firstly, they demonstrate the advances that have been made in the AI’s ability to parse and synthesize clinical information in a manner that resonates with established medical assessment tools. Secondly, they also point to the potential for AI to offload certain tasks from the clinician, such as pre-charting and initial decision-making steps, thereby saving time and allowing for a more efficient patient-clinician interaction.

Nevertheless, while AI demonstrates a level of competence in clinical performance, it is essential to recognize the intrinsic value of the physician’s expertise, particularly in interpreting complex clinical scenarios and making nuanced judgments that AI cannot yet replicate. The performance of ChatGPT 4.0 in these structured assessments can serve as a benchmark for further development, with the aim to enhance its capabilities in more complex and less predictable clinical situations.

### 4.2. Contextualization with Previous Research

Our study’s findings echo the growing body of literature that recognizes the promising role of AI in healthcare, particularly in the field of telemedicine [19,20]. Previous research has consistently indicated the potential for AI to support clinical decision-making [21], reduce physician burnout [22], and improve patient outcomes through enhanced diagnostic and prognostic tools [23]. However, these studies also reflect the understanding that AI should augment rather than replace human clinicians [24]. Notably, comparisons with our findings reveal a consistent theme: the performance of AI in healthcare settings is highly reliant on the quality of the data it is trained on and its ability to learn from diverse clinical encounters [25].

Our study distinguishes itself by being one of the first to examine the specific application of ChatGPT 4.0 in the telemedicine management of metastatic prostate cancer, offering new insights into the capabilities of advanced AI models in a highly specialized domain of medicine [26]. The unique contributions of this research lie in its detailed analysis of AI’s concordance with oncologists’ decisions, a critical exploration that draws upon a diverse array of studies [27,28,29,30,31,32]. Doyle et al. [27] provide foundational insights into the cascaded discrimination of histopathological classes, emphasizing the potential for AI in enhancing diagnostic accuracy in prostate cancer. Similarly, the work by Bulten et al. [28] demonstrates the capabilities of automated deep-learning systems for Gleason grading, showcasing the significant advancements AI brings to the precision and reliability of prostate cancer diagnostics. Kott et al. [29] further the discussion with their development of a deep learning algorithm specifically tailored for the histopathologic diagnosis and Gleason grading of prostate cancer biopsies, highlighting the innovative approaches being taken in the field. Mosquera-Lopez et al. [30] offer a comprehensive review of texture-based systems for computer-aided prostate cancer diagnosis, underlining the importance of sophisticated image analysis techniques. Karimi et al. [31] and Nagpal et al. [32] both contribute critical viewpoints on the role of multiscale decision aggregation and data augmentation in deep learning-based Gleason grading, affirming the transformative impact of these methodologies on the path towards more accurate and nuanced AI-assisted diagnostic processes.

These results demonstrate AI’s utility in real-world medical settings and offer a glimpse into a future where AI could meaningfully alleviate the growing pressures on healthcare systems. The prior work of Goldenberg et al. [33] catalyzed a transformative integration of AI into the management of prostate cancer, heralding an innovative era in medical practice. Building on this pivotal foundation, Tătaru et al. [34] comprehensively delineated AI’s role throughout the continuum of prostate cancer care. George et al. [35] further elucidated the capacity of AI to revolutionize oncological outcomes, harnessing unprecedented precision and innovation. Steiner et al. [36] explored the synergistic potential between AI and pathologists to refine the processes of reviewing and grading prostate biopsies. Marginean et al. [37] showcased the automation and standardization of Gleason grading, a critical diagnostic tool, through advanced AI applications. Ström et al. [38] underscored the substantial enhancements AI brings to diagnostic accuracy and patient management strategies. Additionally, a recent study by Kufel et al. [39] highlighted the integration of AI-driven tools in radiology and diagnostic imaging, examining ChatGPT’s performance on the Polish specialty exam in radiology. This study identified strengths and limitations of the model, demonstrating that while ChatGPT did not pass the exam, it performed closely in certain categories, indicating potential for improvement and further application in medical education and diagnostics. Collectively, these seminal works underscore AI’s transformative influence on the diagnostic and therapeutic landscapes of prostate cancer, significantly advancing healthcare delivery and improving patient outcomes.

ChatGPT has shown significant potential in various healthcare applications, offering innovative solutions across multiple domains. In clinical decision support, ChatGPT aids healthcare professionals by providing evidence-based recommendations and generating clinical notes, thereby enhancing workflow efficiency and accuracy. For example, it can assist in diagnosing conditions by synthesizing patient data and suggesting possible diagnoses and treatment options, effectively acting as a second opinion for clinicians [40]. Additionally, ChatGPT’s capabilities extend to personalized patient engagement, where it can deliver tailored health reminders, appointment notifications, and follow-up care instructions, improving patient adherence and outcomes [41].

In medical education, ChatGPT supports students and professionals by providing instant access to a vast array of medical knowledge, facilitating learning through interactive Q&A sessions, and even helping in preparing for exams like the USMLE [42]. Moreover, its utility in research is notable, as it can expedite literature reviews, generate research hypotheses, and assist in drafting and editing scholarly articles [43]. Despite these advantages, challenges such as the potential for generating incorrect information and perpetuating biases inherent in training data remain critical concerns that need addressing to maximize its effectiveness and reliability in real-world applications [44].

### 4.3. Strengths and Limitations

One of the key strengths of our study lies in its innovative approach, combining telemedicine and AI to manage metastatic prostate cancer. The use of real-world clinical scenarios to assess the performance of ChatGPT 4.0 against established medical oncologists adds practical relevance to our findings. Additionally, the employment of standardized assessment tools like miniCEX and MDM lends credibility to our comparative results. Furthermore, the study benefits from a clearly defined patient cohort, comprehensive baseline characteristic data, and a significant sample size for this niche of telemedicine research, enhancing the generalizability of the findings.

Despite these strengths, our study has limitations that must be acknowledged. The retrospective nature of the study may introduce biases related to data collection and patient selection. The use of a single AI tool, ChatGPT 4.0, may not represent the performance of other AI systems available, limiting the breadth of our conclusions.

Another limitation is the focus on a single disease entity, which may not fully demonstrate the versatility of AI in managing various medical conditions. Additionally, the moderate concordance rate suggests there is room for improvement in AI’s decision-making capabilities, which could be addressed by incorporating more advanced machine learning techniques and larger datasets for training.

The reliance on documented medical records also assumes the completeness and accuracy of these records, which may not always capture the nuances of patient care and decision-making. Lastly, while our study provides insight into AI’s potential, it does not fully explore the impact on patient outcomes, which is a critical measure of success in healthcare.

To broaden the scope and depth of understanding the role of AI in telemedicine, several avenues for future research emerge from the limitations of the current study. Firstly, the initiation of prospective multicenter trials could be invaluable in confirming the initial findings and exploring their applicability across varied populations and healthcare systems. This would help in understanding how different demographic, socioeconomic, and healthcare infrastructure variables affect AI’s performance.

Further comparative studies are also critical, especially those that evaluate a range of AI platforms to determine the breadth of AI’s utility in telemedicine. Such studies would contribute to a more comprehensive understanding of which AI characteristics are most beneficial in clinical settings. Moving beyond metastatic prostate cancer to include a spectrum of diseases could reveal the adaptability and robustness of AI in handling diverse clinical scenarios, potentially illuminating the areas where AI assistance is most effective.

Moreover, the incorporation of patient-reported outcomes in future research would bridge the gap between clinical decision-making and patient satisfaction. It would provide insight into how the integration of AI in telemedicine influences aspects of care that matter to patients, such as quality of life and perceived efficacy of care.

### 4.4. Conclusions

These findings suggest that while ChatGPT can effectively mimic some aspects of oncologist decision-making, the degree to which it aligns with human experts is not absolute. The moderate concordance underpins the potential utility of AI in supporting clinical decision-making but also highlights the necessity for continued supervision and final decision-making by healthcare professionals. Moreover, these results indicate that ChatGPT could serve as a valuable adjunct tool in the telemedicine management of mPC, providing pre-charting assistance and preliminary decision-making support that can augment the efficiency and possibly the efficacy of patient care. However, the discrepancies noted in the study also underscore the importance of further refinement of AI algorithms to improve alignment with clinical practice patterns.

### 4.5. Future Directions

The findings of this study highlight several promising avenues for future research to further explore the potential and limitations of AI tools like ChatGPT 4.0 in telemedicine. Firstly, prospective multicenter trials should be initiated to validate the initial findings of this study and to assess the generalizability of ChatGPT’s performance across diverse healthcare settings and patient populations. These trials could provide insights into how demographic, socioeconomic, and healthcare infrastructure variations impact the effectiveness of AI in telemedicine.

Expanding the scope of research beyond metastatic prostate cancer to include a broader spectrum of diseases would be essential in evaluating the versatility and adaptability of AI in handling various clinical scenarios. Such studies could help identify the conditions where AI assistance is most beneficial and uncover any specific challenges associated with different types of diseases.

Moreover, future research should focus on enhancing the AI models by training them with larger and more diverse datasets. This could improve the accuracy and reliability of AI tools in clinical decision-making. Incorporating real-time learning capabilities where the AI can continuously update its knowledge base with new medical findings and patient data could also be explored.

Another critical area for future investigation is the integration of patient-reported outcomes in evaluating the effectiveness of AI in telemedicine. Understanding how AI influences patient satisfaction, quality of life, and perceived efficacy of care will be vital in assessing its overall impact on patient-centered care.

Additionally, exploring the integration of AI tools like ChatGPT with other digital health technologies, such as wearable devices and remote monitoring systems, could provide a more comprehensive approach to patient management. This integration could enhance the continuous monitoring and personalized care for patients with chronic conditions, further reducing the need for frequent in-person visits.

Assessing the cost-effectiveness of implementing AI in telemedicine and its impact on healthcare resource utilization is another important direction. Such studies could provide valuable insights for policymakers and healthcare providers considering the adoption of AI technologies, highlighting potential savings and efficiencies gained from AI-assisted telemedicine.

Finally, addressing the ethical and regulatory challenges associated with the use of AI in healthcare is crucial. Future research should explore frameworks for ensuring patient data privacy, consent, and the transparency of AI decision-making processes. This will be essential in building trust among patients and healthcare providers and ensuring the responsible deployment of AI technologies in clinical practice.

## Figures and Tables

**Table 1:** Patients’ baseline characteristics (*n* = 102).

Characteristics	
Median Age (Range), Years	75 (53–99)
Race, *n* (%)	
White	99 (97.06)
Hispanic	2 (1.96)
Hawaiian or Pacific Islander	1 (0.98)
Median Gleason Score (Range)	7 (6–10)
Sites of Metastasis, *n* (%)	
Bone	73 (47.40)
Lymph Node	68 (44.16)
Lung	9 (5.84)
Liver	4 (2.60)
ECOG Performance Status, *n* (%)	
0	0
1	25 (26.88)
>1	51 (54.84)
Coexisting Conditions, *n* (%)	
Diabetes Mellitus	19 (11.11)
Hypertension	51 (28.92)
Hyperlipidemia	42 (25.56)
Depression	13 (7.60)
Gastroesophageal Reflux Disease	31 (18.13)
Atrial Fibrillation	11 (6.43)
Heart Failure	4 (2.34)

**Table 2:** MiniCEX score by ChatGPT and MDM length of note written by ChatGPT.

	Median	Standard Deviation	Min, Max
MiniCEX score	8	0.59	7, 9
MDM length	41	6.06	24, 72

## Data Availability

The data that support the findings of this study are available on request from the corresponding author (U.S.). The data are not publicly available due to them containing information that could compromise research participant privacy.
